# Study of whole genome linkage disequilibrium patterns of Iranian water buffalo breeds using the Axiom Buffalo Genotyping 90K Array

**DOI:** 10.1371/journal.pone.0217687

**Published:** 2019-05-31

**Authors:** Mahdi Mokhber, Mohammad Moradi Shahrbabak, Mostafa Sadeghi, Hossein Moradi Shahrbabak, Alessandra Stella, Ezequiel Nicolzzi, John L. Williams

**Affiliations:** 1 Department of Animal Science, Faculty of Agriculture, Urmia University, Urmia, Iran; 2 Department of Animal Science, Faculty of Agricultural Science and Engineering, University College of Agriculture and Natural Resources (UTCAN), University of Tehran, Karaj, Iran; 3 Fondazione Parco Tecnologico Padano, Lodi, Italy; 4 Davies Research Centre, School of Animal and Veterinary Sciences, University of Adelaide, Roseworthy, South Australia, Australia; National Cheng Kung University, TAIWAN

## Abstract

Accuracy of genome-wide association studies, and the successful implementation of genomic selection depends on the level of linkage disequilibrium (LD) across the genome and also the persistence of LD phase between populations. In the present study LD between adjacent SNPs and LD decay between SNPs was calculated in three Iranian water buffalo populations. Persistence of LD phase was evaluated across these populations and effective population size (Ne) was estimated from corrected r^2^ information. A set of 404 individuals from three Iranian buffalo populations were genotyped with the Axiom Buffalo Genotyping 90K Array. Average r^2^ and |D'| between adjacent SNP pairs across all chromosomes was 0.27 and 0.66 for AZI, 0.29 and 0.68 for KHU, and 0.32 and 0.72 for MAZ. The LD between the SNPs decreased with increasing physical distance from 100Kb to 1Mb between markers, from 0.234 to 0.018 for AZI, 0.254 to 0.034 for KHU, and 0.297 to 0.119 for MAZ, respectively. These results indicate that a density of 90K SNP is sufficient for genomic analyses relying on long range LD (e.g. GWAS and genomic selection). The persistence of LD phase decreased with increasing marker distances across all the populations, but remained above 0.8 for AZI and KHU for marker distances up to 100Kb. For multi-breed genomic evaluation, the 90K SNP panel is suitable for AZI and KHU buffalo breeds. Estimated effective population sizes for AZI, KHU and MAZ were 477, 212 and 32, respectively, for recent generations. The estimated effective population sizes indicate that the MAZ is at risk and requires careful management.

## Introduction

The water buffalo (*B*. *bubalis*) is an important livestock resource in many regions of the world, particularly in tropical and subtropical countries. Water buffalo produce milk and meat, and are used as draught animals in developing countries [1,2]. There are two types of domestic water buffalo. The river buffalo, which originated in the Indian sub-continent and are now spread widely from India to Europe. The swamp buffalo, which originated in Northern Thailand or Laos, is the most common buffalo type in Asia, from India to the Philippines. Water buffalo and cattle (*Bos taurus*) belong to the sub-family, Bovinae. While cattle were domesticated between 8000 and 10,000 years ago [3], domestication of river and swamp buffalo was more recent and has been estimated to have been between 5000 and 7000 years ago [2,4,5].

Accuracy of genome-wide association studies, and also genomic selection, is dependent on the level of LD across the genome [6], which is influenced by population history, the breeding systems used, e.g. natural mating or artificial insemination, and admixture among populations [7]. Therefore, an LD map of the species is a fundamental tool for the application of genetic selection to improve economically important traits [8]. Information on genome-wide LD is also essential for choosing SNP to locate QTL in a genome wide association studies [8–10], for the investigation of the diversity among breeds [6], to trace selective sweeps [10,11] and to assess the distribution of recombination events [6]. Population demography may also be studied based on LD information, e.g. to assess the changes in the effective population size through generations [9]. Methods for estimating the effective population size (Ne) are either demographic, pedigree-based, of marker-based [12]. Marker-based methods to estimate Ne use information extracted from genetic data, such as heterozygosity excess, LD, changes in allele frequency, and amount of genetic variation within and between populations [13].

LD has been widely studied in various domestic species [6,8,14–19]. The two most commonly used measures to evaluate LD, for bi-allelic markers, are r^2^ and |D'| [6,20]. The r^2^ value is the correlation between two loci [21] and is preferred for association studies, because there is a simple inverse relationship between r^2^ and the sample size that is required to discover associations between a QTL and SNP [22]. |D'| varies between 0 and 1: values below 1 indicate recombination between two loci, while a value of 1 indicates lack of recombination between two loci. The accuracy of estimating |D'| depends on sample size and allele frequency [6], and is severely inflated for small sample sizes and in the presence of rare alleles [23]. Calculating r^2^ is much less affected by low allele frequencies and small sample size [24,25].

The level and pattern of LD observed in a population is influenced by factors such as; the sub-division and admixture of populations [24], genetic bottlenecks [26], genetic drift, inbreeding, recombination rate, gene conversion [27,28] and selection [27–29]. Persistence of LD phase can be used to trace history of a species and relationships among individuals within that species [30]. The extent and persistence of LD in livestock [25,31–33] is much higher than that found in human populations [28], because genetic selection and breeding methods tend to reduce the effective population size [34].

The objectives of this study were to assess: (i) LD between adjacent SNPs and LD decay according to physical distance between bi-allelic SNPs in three Iranian indigenous water buffalo populations using the statistics (*r*^2^) and |D'|; (ii) the consistency of the LD phase across studied populations; and (iii) the effective population size in relation to LD decay.

## Materials and methods

### Animal and DNA samples

Selection of animals and collection of samples for Azeri (AZI) and Khuzestani (KHU) breeds is described in Mokhber et al. [35]. Samples from the Mazandarani (MAZ) breed were collected from the Miankaleh wildlife sanctuary of Mazandaran province (36.81° N, 53.41°E), located in the northern part of Iran (S1 Fig) [36]. The majority of MAZ buffaloes (about half of the living MAZ buffaloes) are raised at Miankaleh. The MAZ buffaloes outside of the Miankaleh are mostly raised at Golestan province and were not sampled.

### Genotyping and data quality control

Genomic DNA was extracted from blood by the modified salting out method [37] and from hair samples as described by Alberts et al. [38]. The set of 412 water buffalo samples from AZI (n = 262), KHU (n = 123) and MAZ (n = 27) were genotyped using the Axiom Buffalo Genotyping 90K Array. Genotyping was carried out by Affymetrix (Sana Clara, Ca, USA). SNP genotypes were extracted from raw data using the AffyPipe workflow [39]. The genotypes for each population were filtered for quality separately, using PLINK software [40]. Single nucleotide polymorphisms (SNPs) with minor allele frequencies (MAF) below 0.05, SNPs with call rate below 0.05 or which were not in Hardy-Weinberg Equilibrium (P-value >10e^-6^) were removed. Individuals with more than 5% missing genotypes, were excluded from data set. After quality control for each population, genotypes from the three breeds were merged into a single file, and SNPs that were common across all three populations were retained for further analyses. The Axiom Buffalo Genotyping 90K Array was designed based on alignment of buffalo sequences to the bovine UMD3.1 genome assembly [41], therefore this bovine reference genome sequence and relative bovine positions were used in the present study.

### Measures of linkage disequilibrium

The LD between two SNPs was evaluated using the statistics r^2^ [20] and the absolute D-value (|D'|) [42], which were calculated as follows:

$$r^{2}\;=\;\frac{{\left(D\right)}^{2}}{{\left(freq\;A*freq\;a*freq\;B*frq\;b\right)}^{}}$$

Where

$$D^{}\;=\;freq\;AB-freq\;A*freq\;B$$

And

D'={Dmin(freqA*freqb,freqB*frqa)ifD>0Dmin(freqA*freqB*freqa*frqb)ifD<0

Where SNP pairs had alleles *A* and *a* at the first locus and *B* and *b* at the second locus, *freq A*, *freq a*, *freq B and freq*.*b* denote frequencies of alleles *A*, *a*, *B*, and *b*, respectively, and *freq AB* denote frequency of haplotype *AB* in the population. The r^2^ and |D'| were calculated between adjacent markers and SNP pairs with physical distances from 0 to 15 Mb for each population, using *SnppLD* software (Sargolzaei M, University of Guelph, Canada) [25].

The average r^2^ and |D'| of adjacent SNPs were estimated for each chromosome. SNP pairs were grouped by their pairwise physical distance, based on their position in the UMD3.1 reference cattle sequence, into intervals of 100 Kb (from 0 to 15 Mb). Average r^2^ for SNP pairs in each interval was estimated [32]. The consistency of LD between populations was measured by the correlation of the root of r^2^ of adjacent marker pairs on each chromosome [32]. The consistency of LD phase between two populations was measured by persistence of phase. The correlation of LD between two populations A and B for a common set of markers was calculated as [32]:

$$r_{ij}\;=\;\frac{\sum_{(i,j)\;}^{}\left(r_{ij(A)}-{\overset{-}{r}}_{A})(r_{ij(B)}-{\overset{-}{r}}_{B}\right)}{S_{A}S_{B}}$$

Where *r*_*ij*_ is the correlation of phase between *r*_*ij(A)*_ in population A and *r*_*ij(B)*_ in population B, *S*_*A*_ and *S*_*B*_ are the standard deviation of *r*_*ij(A)*_ and *r*_*ij(B)*_ respectively, and *r*_*A*_ and *r*_*B*_ are the average *r*_*ij*_ across all SNP *i* and *j* within the common set of markers.

### Estimation of historical effective population size

The historical effective population size for AZI, KHU and MAZ was calculated for t generations in the past as follows:

$$Ne\;=\;(\frac{1}{4c})(\frac{1}{r^{2}}-1)$$

[43],

where Ne is the effective population size, c is the genetic distance between the SNPs in Morgans. The physical distances between SNPs were converted to genetic distances using the approximation 1 cM~1 Mb for all the chromosomes [19,44]. r^2^ is the average corrected r^2^ value at a given distance. A sample size correction was carried out for all of the computed r^2^ values using the following equation

$$Corrected\;r^{2}\;=\;\frac{Computed\;r^{2}-\frac{1}{n}}{1-\frac{1}{n}}$$

[19]

where, n is the number of haplotypes in the sample. It should be noted, the estimated Ne value is infinite at r^2^ = 0 and zero at r^2^ = 1. Therefore. Only values of r^2^ between 0.01 and 0.99 were used to estimate Ne.

The generation of Ne for a given distance was estimated by:

$$t\;=\;\frac{1}{2c}$$

[45],

where *t* was calculated for the corresponding genetic distance (c) in intervals of 100 Kb (from 0 to 15 Mb). The historical Ne was investigated at 150 time points from recent to 500 generations in the past.

## Results

### SNP frequency and distribution

After quality control for each population, a set of 63824, 62667 and 58588 SNPs remained for AZI, KHU and MAZ breeds, respectively. SNPs that were in common across all breeds, were merged in single file. The final data set comprised 57212 SNPs from 396 individuals (253 AZI and 118 KHU and 25 MAZ) which was used for further analyses. After removing SNP with a MAF less than 0.05, the mean MAF observed in the Iranian populations was 0.333, 0.321 and 0.299 for AZI, KHU and MAZ, respectively, for the common SNP set. As the buffalo genome available is highly fragmented SNPs were mapped to the bovine sequence (version Btau UMD3.1). A summary of SNP numbers for each bovine chromosome with MAF in each population is shown in S1 Table.

Distribution of SNPs with the distance between adjacent SNPs as mapped to the bovine genome sequence (version Btau UMD3.1), is shown in S2 Table. Distances between 93% of adjacent SNPs were less than 100 Kb, while the distances between 60% of adjacent SNPs were between 20-40Kb for all of the three breeds (S2 Table.).

The average estimated physical distance between adjacent markers in the common set was 46 Kb, and covered 2.65 Gb of total genome length (Table 1). The highest number of polymorphic SNPs was on BTA1 (N = 3549) and the lowest on BTA 27 (N = 1004). The longest interval between polymorphic SNP was 2461.72 Kb on BTA10 and the shortest was 0.008 Kb on BTA15.

**Table 1:** Distance and linkage disequilibrium (r^2^ and |D'|) between adjacent polymorphic SNP and consistency of r^2^ between breeds based on Bos Taurus chromosome (BTA).

Chr	Number of SNP	Mean Distance (Kb)	MinDistance (Kb)	Max Distance (Kb)	Length (Mb)	AZI_ (Mean± SD)		KHU__ (Mean± SD)		MAZ__ (Mean± SD)		Consistency^1^		
						r^2^	|D'|	r^2^	|D'|	r^2^	|D'|	AZI and KHU	AZI and MAZ^2^	KHU and MAZ
1	3549	44.6	211.69	0.027	158.1	0.28±0.29	0.67±0.32	0.29±0.29	0.69±0.32	0.32±0.32	0.71±0.31	0.83	0.61	0.58
2	2997	45.5	414.07	0.629	136.5	0.28±0.29	0.66±0.33	0.30±0.31	0.69±0.32	0.33±0.32	0.72±0.31	0.82	0.63	0.56
3	2680	45.2	471.66	0.137	121.2	0.28±0.29	0.66±0.32	0.31±0.31	0.70±0.32	0.33±0.32	0.72±0.32	0.84	0.61	0.56
4	2677	45.0	292.12	0.062	120.4	0.28±0.28	0.66±0.33	0.29±0.30	0.69±0.32	0.32±0.32	0.70±0.32	0.83	0.61	0.58
5	2607	46.2	552.62	0.279	120.5	0.29±0.30	0.67±0.33	0.31±0.31	0.70±0.32	0.34±0.32	0.73±0.30	0.86	0.62	0.57
6	2599	45.9	929.84	0.503	119.4	0.27±0.28	0.65±0.33	0.29±0.29	0.69±0.32	0.33±0.32	0.71±0.31	0.84	0.63	0.60
7	2456	45.8	1101.73	0.201	112.4	0.26±0.29	0.62±0.34	0.28±0.30	0.66±0.32	0.30±0.31	0.69±0.31	0.83	0.63	0.57
8	2405	47.1	517.16	0.404	113.2	0.29±0.30	0.68±0.33	0.31±0.31	0.70±0.32	0.32±0.31	0.72±0.30	0.85	0.60	0.58
9	2304	45.8	384.01	0.708	105.5	0.26±0.28	0.65±0.33	0.28±0.29	0.68±0.32	0.30±0.30	0.72±0.31	0.84	0.62	0.59
10	2222	46.7	2461.72	1.553	103.9	0.28±0.29	0.65±0.33	0.29±0.30	0.67±0.32	0.35±0.33	0.73±0.30	0.83	0.57	0.53
11	2353	45.5	395.13	0.135	107.1	0.27±0.29	0.66±0.33	0.29±0.30	0.68±0.32	0.35±0.33	0.74±0.30	0.84	0.60	0.57
12	1881	48.4	1731.46	0.617	91.0	0.27±0.28	0.66±0.33	0.28±0.29	0.67±0.32	0.32±0.32	0.72±0.31	0.83	0.63	0.60
13	1880	44.5	634.95	1.086	83.7	0.25±0.27	0.64±0.33	0.27±0.29	0.66±0.33	0.31±0.31	0.71±0.31	0.83	0.59	0.56
14	1893	44.0	414.42	0.112	83.3	0.26±0.27	0.65±0.33	0.29±0.29	0.68±0.32	0.32±0.31	0.73±0.31	0.83	0.58	0.53
15	1845	46.0	1212.36	0.008	84.8	0.26±0.27	0.65±0.33	0.27±0.29	0.67±0.32	0.32±0.32	0.72±0.31	0.82	0.67	0.60
16	1737	46.7	1310.90	0.027	81.2	0.29±0.30	0.67±0.33	0.29±0.29	0.69±0.32	0.34±0.33	0.73±0.31	0.84	0.63	0.60
17	1621	46.3	573.05	1.913	75.0	0.28±0.30	0.66±0.34	0.30±0.31	0.69±0.33	0.32±0.31	0.71±0.31	0.85	0.61	0.59
18	1417	46.3	721.85	0.086	65.6	0.24±0.26	0.63±0.33	0.26±0.29	0.67±0.33	0.31±0.31	0.72±0.3	0.82	0.58	0.55
19	1409	45.1	431.85	2.880	63.5	0.27±0.29	0.66±0.34	0.28±0.28	0.68±0.32	0.33±0.32	0.74±0.29	0.80	0.63	0.56
20	1559	46.0	310.67	1.589	71.8	0.27±0.28	0.64±0.33	0.28±0.30	0.66±0.33	0.30±0.30	0.71±0.30	0.83	0.63	0.58
21	1512	45.7	394.36	2.625	69.1	0.27±0.29	0.65±0.33	0.28±0.30	0.67±0.33	0.34±0.33	0.72±0.32	0.84	0.65	0.60
22	1400	43.7	381.49	0.651	61.2	0.25±0.29	0.63±0.34	0.26±0.29	0.65±0.33	0.32±0.33	0.71±0.31	0.84	0.61	0.56
23	1152	45.2	1154.70	0.291	52.0	0.27±0.29	0.65±0.34	0.29±0.30	0.68±0.33	0.31±0.31	0.69±0.32	0.84	0.62	0.57
24	1443	43.0	236.15	0.014	62.0	0.25±0.26	0.64±0.34	0.27±0.28	0.68±0.33	0.33±0.32	0.72±0.32	0.82	0.56	0.51
25	1053	40.5	216.00	0.688	42.6	0.23±0.27	0.62±0.34	0.26±0.29	0.65±0.34	0.31±0.32	0.71±0.32	0.84	0.60	0.58
26	1154	44.3	256.76	0.489	51.1	0.24±0.26	0.62±0.33	0.27±0.29	0.65±0.33	0.30±0.31	0.70±0.31	0.81	0.56	0.52
27	1004	45.1	619.59	0.162	45.3	0.24±0.26	0.62±0.33	0.26±0.28	0.67±0.32	0.28±0.30	0.69±0.32	0.78	0.52	0.49
28	1045	44.1	796.62	0.141	46.0	0.27±0.28	0.65±0.33	0.28±0.3	0.66±0.33	0.31±0.31	0.71±0.30	0.83	0.61	0.58
29	1108	45.9	922.65	1.623	50.8	0.24±0.27	0.62±0.34	0.25±0.28	0.65±0.32	0.29±0.30	0.67±0.32	0.82	0.64	0.57
30	2250	66.1	1880.77	0.479	148.7	0.39±0.36	0.74±0.33	0.42±0.38	0.77±0.32	0.41±0.35	0.76±0.29	0.88	0.66	0.62
**Total**	57212	-	-	-	2647.0	-	-	-	-	-	-			
**Mean**	-	46.0	-	-		0.27±0.33	0.66±0.33	0.29±0.32	0.68±0.32	0.32±0.31	0.72±0.31	0.83	0.61	0.57

^1^-The correlation of r^2^ of adjacent SNP pairs between populations

^2^- Azeri and Mazandarani

### Linkage disequilibrium

#### Linkage disequilibrium and consistency of LD between adjacent SNP

LD was calculated separately for each of the three Iranian buffalo breeds using r^2^ and |D’| statistics. Average r^2^ and |D’| between adjacent SNP pairs were 0.27 and 0.66 for AZI, 0.29 and 0.68 for KHU, and 0.32 and 0.72 for MAZ (see Table 1). The proportion of r^2^ values higher than 0.2 and 0.3 were 44.7 and 34.8% for AZI, 46.7 and 36% for KHU, 50.8 and 40.1% for MAZ, respectively (S3 Table). The correlation of LD between the AZI and KHU breeds was 0.83 (ranging from 0.78 to 0.88 across all chromosomes) which was higher than the correlation between AZI and KHU (0.61), and KHU and MAZ (0.57) (Table 1 and S2 Fig).

#### Linkage disequilibrium decay and persistence of LD phase

**Fig 1:**
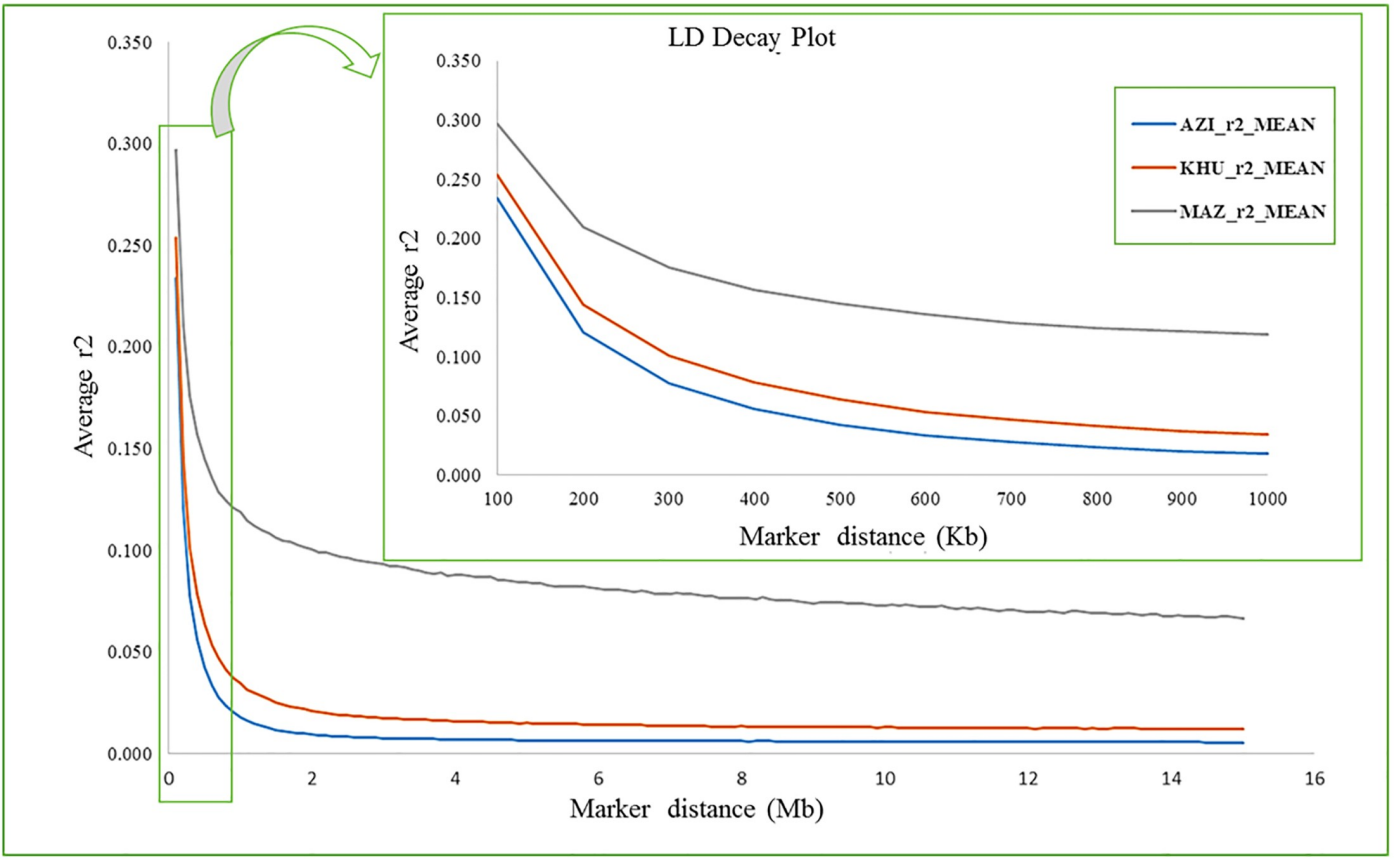
Average LD decay over physical distance For AZI, KHU and MAZ buffalo breeds.

**Fig 2:**
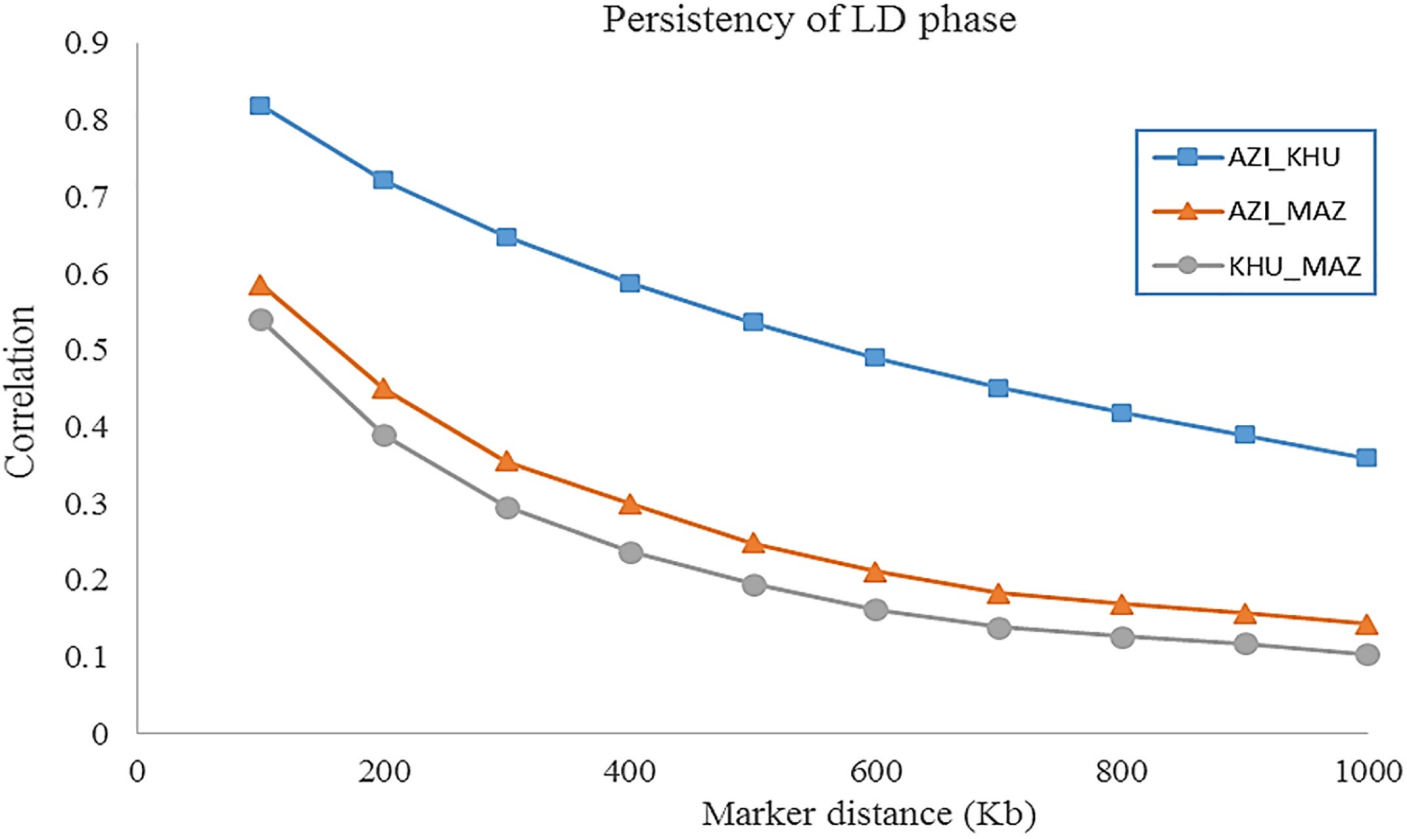
Consistency of gametic phase at given distances for AZI and KHU, AZI and MAZ, and KHU and MAZ buffalo breed pairs.

The average decay of LD over physical distance was calculated by chromosome (S3–S5 Figs), and the overall genome LD was also calculated for each breed for an averaged interval of 100 Kbp (Fig 1 and S4 Table). Comparing the different breeds, the LD was highest for MAZ and lowest for AZI for all SNP distances. The patterns of LD decay was similar for AZI and KHU but differed in MAZ. As expected, the persistence of LD phase decreased with increasing physical distance between markers for all breeds (S5 Table). This decrease was rapid for distances shorter than 300 Kb, while the reduction in LD for distances of 1Mb to 15Mb was very small. At all intervals, the highest correlation was between AZI and KHU and the lowest was between KHU and MAZ. For distances below 100Kb (with an average of 56.9 Kb), the correlation varied from 0.82 for AZI and KHU to 0.54 for KHU and MAZ. While for distances greater than 1Mb the correlations varied from 0.36 for AZI and KHU to 0.10 for KHU and MAZ (Fig 2 and S5 Table). At all distances between SNP, average LD was highest for MAZ, intermediate for KHU and lowest for AZI.

### Effective Population Size (Ne) based on genomic data

**Fig 3:**
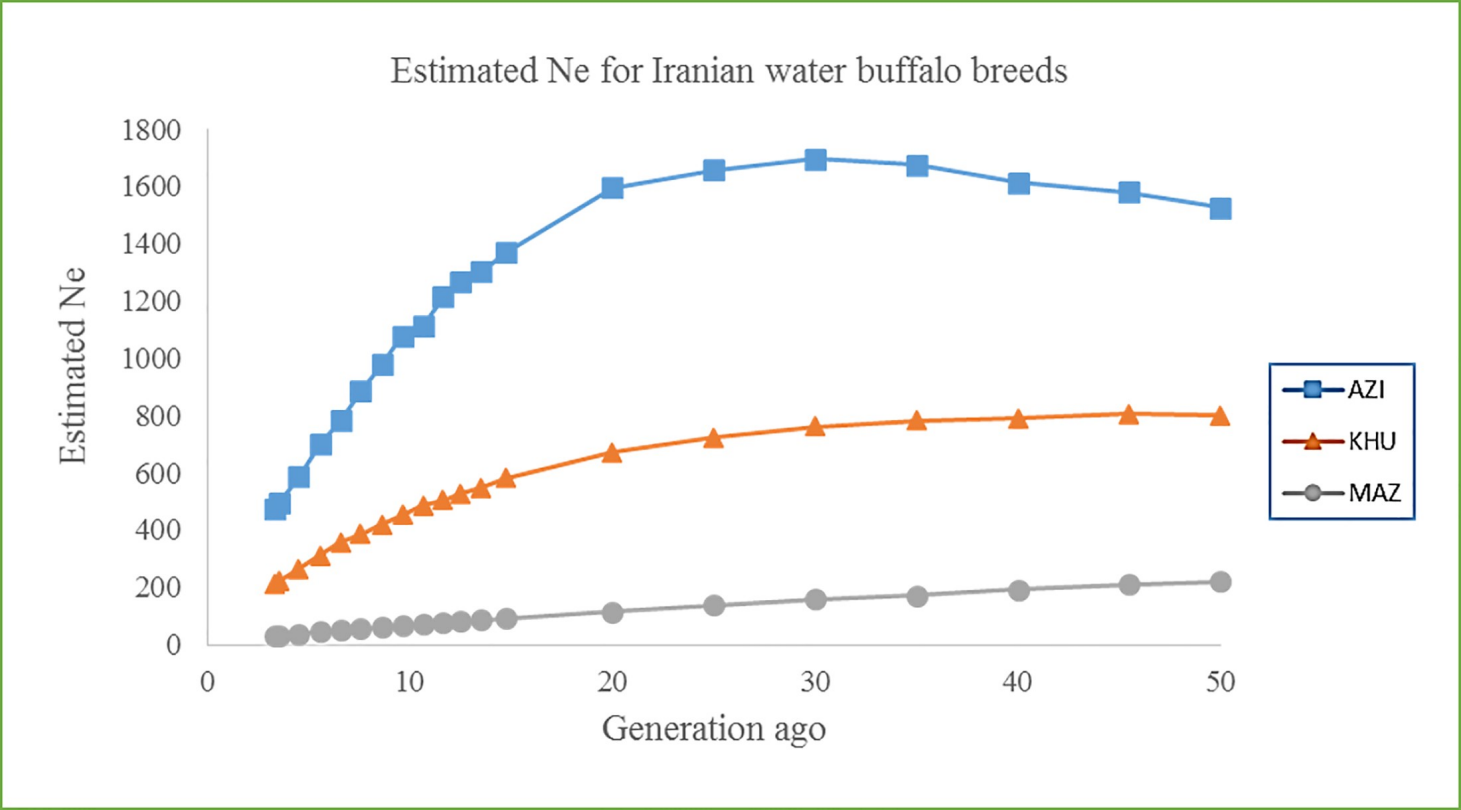
Past effective population size (Ne) over generations based on linkage disequilibrium calculations all genome. The Ne from 500 to 3 generations ago.

In the absence of pedigree information, analysis of LD can be used to estimate the effective population size, Ne [12]. LD between SNPs that are close together reveals historic events, while LD between more distant SNPs can be used to explore more recent population history. Ne in the recent generations was estimated to be 477, 212 and 32 for AZI, KHU and MAZ, respectively. While for 500 generation ago, Ne was estimated as 826, 748 and 632 for AZI, KHU and MAZ, respectively (Fig 3 and S6 Table). However, changes were not linear, and the intensity and direction of changes differed over time for each population. The reduction in Ne in AZI and KHU has been rapid over the last 20 generation. Ne for AZI seems to have increased between 100–40 generation ago.

## Discussion

### SNP frequency and distribution

After quality control, at total of 57,212 common SNPs remained across all chromosomes for the three Iranian buffalo breeds, which is comparable with the number of polymorphic SNP found in Brazilian dairy buffaloes [46] but lower than 67,580 polymorphic SNPs, seen in Italian Mediterranean buffalos [47]. As would be expected, the Axiom Buffalo Genotyping 90K Array had more polymorphic SNP in the three Iranian buffalo populations (65–75% polymorphic SNP) than the Bovine HD SNP chip (Illumina, Inc, San Diego, CA, USA), for which only 15,745 of the 777,962 (2%) SNPs on the array were polymorphic in buffalo [48]. After filtering, the average MAF for the SNP on the Buffalo array, in the Iranian breeds, was between 0.29–0.31. This is comparable with the average MAF for SNPs on the Illumina BovineSNP50K BeadChip used routinely for cattle, where the average MAF is between 0.24 and 0.27 in different cattle breeds [6,16,49]. SNP panels with this level polymorphism have been successfully used to explore LD and carry out genome wide associations studies [14].

### Linkage disequilibrium

#### Linkage disequilibrium and consistency of LD between adjacent SNP

The average r^2^ and |D’| between adjacent SNP pairs in the present study were consistent with the values reported by Cardoso et al. for Brazilian dairy buffalos (0.29 and 0.72 for r^2^ and |D'|), using the same SNP set [46]. The average r^2^ and |D’| values between adjacent SNP pairs reported here are similar to those for Holstein cattle [6,14], but higher than those seen for other cattle breeds, eg; composite Brazilian Beef cattle [50] and the Gyr [18]. Breeding strategies and practices for dairy cattle breeds and domestic dairy buffalo are similar, using few bulls, while breeding strategies for other breeds may use a wider genetic pool. McKay et al. [8] reported the average LD between 0.15 to 0.20 for six taurine cattle breeds and two zebu breeds for and inter-marker distance of 100Kb. This compares with LD between 0.20 and 0.26 reported here for buffalo for same distance (S4 Table). The r^2^ and |D’| values are related to breed diversity, such that populations with lower diversity have higher average LD between adjacent loci. It should be noted that the level of LD differs across chromosomes e.g. Nelore cattle genotyped using a high-density bovine SNP marker panel gave a wide range of LD estimates across different chromosome regions, ranging from 0.17 to 0.24 for r^2^ and from 0.55 to 0.72 for |D'| [51]. These differences can be attributed to variable recombination rates between and within chromosomes, heterozygosity, genetic drift and effects of selection [9]. In designing marker panels for particular populations the density of loci could be varied depending on the level of LD at specific regions of the genome to optimize the information recovered.

The consistency of LD is high between the AZI and KHU breeds, indicating the close genetic relation of these breeds, whereas the comparison of AZI with MAZ, and KHU with MAZ show lower preservation of LD. This is consistent with Colli et al [52] who reported the lowest differentiation between AZI and KHU (0.021), moderate for AZI and MAZ (0.038), and highest for KHU and MAZ (0.045).

#### Linkage disequilibrium decay and persistence of LD phase

The level of LD between markers is important in the design and success of genome wide association studies and genomic selection [53]: genomic breeding estimates are more accurate when the mean r^2^ between adjacent SNPs is higher, as the makers are more likely to predict the alleles at adjacent QTLs. Marker spacing giving an r^2^ of at least 0.2 is recommended to estimate genomic breeding values [27,53,54] while a r^2^ of 0.3 and above has been suggested for genome wide association studies and QTL mapping [27]. The average r^2^ values between adjacent markers obtained from using the Axiom Buffalo Genotyping 90K Array were between 0.27 and 0.327 for the populations studied here, which is around the threshold of 0.3.

One of the factors impacting on the accuracy of genomic breeding estimates across-populations is the persistence of LD phase, which reflects the genetic relationship between the populations [55]. The maintenance of LD phase for adjacent SNPs and persistence LD phase between AZI and KHU was higher than AZI and MAZ, and KHU and MAZ at all distances. These results support the AZI and KHU being genetically closer than AZI and MAZ, and KHU and MAZ. The persistence of marker phase between populations decreases as the divergence between the populations increases, and hence a higher marker density is required for more divergent breeds [56]. The present study suggests that the AZI and KHU breeds could be treated as a single population for genomic selection when using the Axiom Buffalo Genotyping 90K Array.

#### Effective population size based on genomic data

Effective populations size (Ne) is related to the history of a population [57] and is a key parameter used in conservation biology [13]. The FAO (1992) reports that with effective population size of 50, the loss of genetic diversity over 10 generations is approximately 10% [58]. LD-based methods with markers spaced at 1Mb tend to overestimate Ne for more than 50 generations ago, while estimates for recent generations are more accurate [12]. Ne estimates presented here are based on corrected r^2^ values, which are less sensitive to allele frequency and a small sample size than |D'| [6]. In the present study, Ne was estimated from SNP distance from recent generation (15Mb) to 500 generation ago (100Kb). The results suggest that Ne has been lower in the recent generations compared with more ancient generations for all three breeds, and that the effective population size is currently highest for AZI and lowest for MAZ. The first buffalo were imported to Iran from India in 2000–2500 BC [59]. The higher Ne estimated for the ancient populations may reflect the diversity in the original Iranian population before it separated into breeds [33]. Artificial insemination has not been widely used in the AZI and KHU populations, which is likely to have contributed to the relative high Ne values for both populations, which are above the threshold (Ne = 100) to ensure that a population is viable for long-term survival [60]. The MAZ population has been geographically isolated and managed in a protected natural area, both of which are contributory factors to the low estimated Ne, which is below 100. Therefore, the MAZ population can be considered as endangered and it is essential to monitor the population and develop a breeding program to ensure viability and avoid inbreeding.

### Conclusions

The average distance between adjacent SNPs in the current Axiom Buffalo Genotyping 90K Array is 30 Kb, based on alignment with the bovine genome. After filtrating for quality and MAF the between-marker distance for the 57212 common SNPs was 46 Kb. The level of LD in Iranian buffalo using this set is above that recommended for genome wide association studies (r^2^> 0.3) or to estimate genomic breeding values (r^2^> 0.2). The calculated Ne from LD decay indicates that the AZI and KHU have a sufficiently large effective population size to be sustainable, while the MAZ has a low effective population and needs careful management to ensure its survival.

## Supporting information

S1 Fig Distributions of the three Iranian buffalo breeds (AZI, KHU and MAZ breeds) used in present study. (TIF)

S2 Fig Consistency of LD phase between AZI and KHU buffalo breeds. (TIF)

S3 Fig LD decay for AZI buffalo breed by chromosome. (TIF)

S4 Fig LD decay for KHU buffalo breed by chromosome. (TIF)

S5 Fig LD decay for MAZ buffalo breed by chromosome. (TIF)

S1 Table Summary of the polymorphic SNP markers and MAF for each chromosome. (XLSX)

S2 Table Distribution of SNP with average distance for all breeds between adjacent SNPs mapped vs the bovine genome sequence (version Btau UMD3.1). (XLSX)

S3 Table Frequency of r2 and |D'| values for AZI, KHU and MAZ buffalo breeds. (XLSX)

S4 Table Average LD decay over physical distance For AZI, KHU and MAZ buffalo breeds. (XLSX)

S5 Table Consistency of gametic phase at given distances for AZI and KHU, AZI and MAZ, and KHU and MAZ buffalo breed pairs. (XLSX)

S6 Table Effective population size for AZI, KHU and MAZ breeds in given number of generations ago. (XLSX)

## Abbreviations

AZI and KHU: Azeri and Khuzestani
AZI and MAZ: Azeri and Mazandarani
AZI: Azeri
KHU and MAZ: Khuzestani and Mazandarani
KHU: Khuzestani
LD: Linkage Disequilibrium
MAF: Minor Allele Frequency
MAZ: Mazandarani
Ne: Effective Population Size
