# Supplementary figures and images for: Study of whole genome linkage disequilibrium patterns of Iranian water buffalo breeds using the Axiom Buffalo Genotyping 90K Array
Source: PLoS One. 2019 May 31;14(5):e0217687. doi: 10.1371/journal.pone.0217687 (PMC6544294; doi:10.1371/journal.pone.0217687)

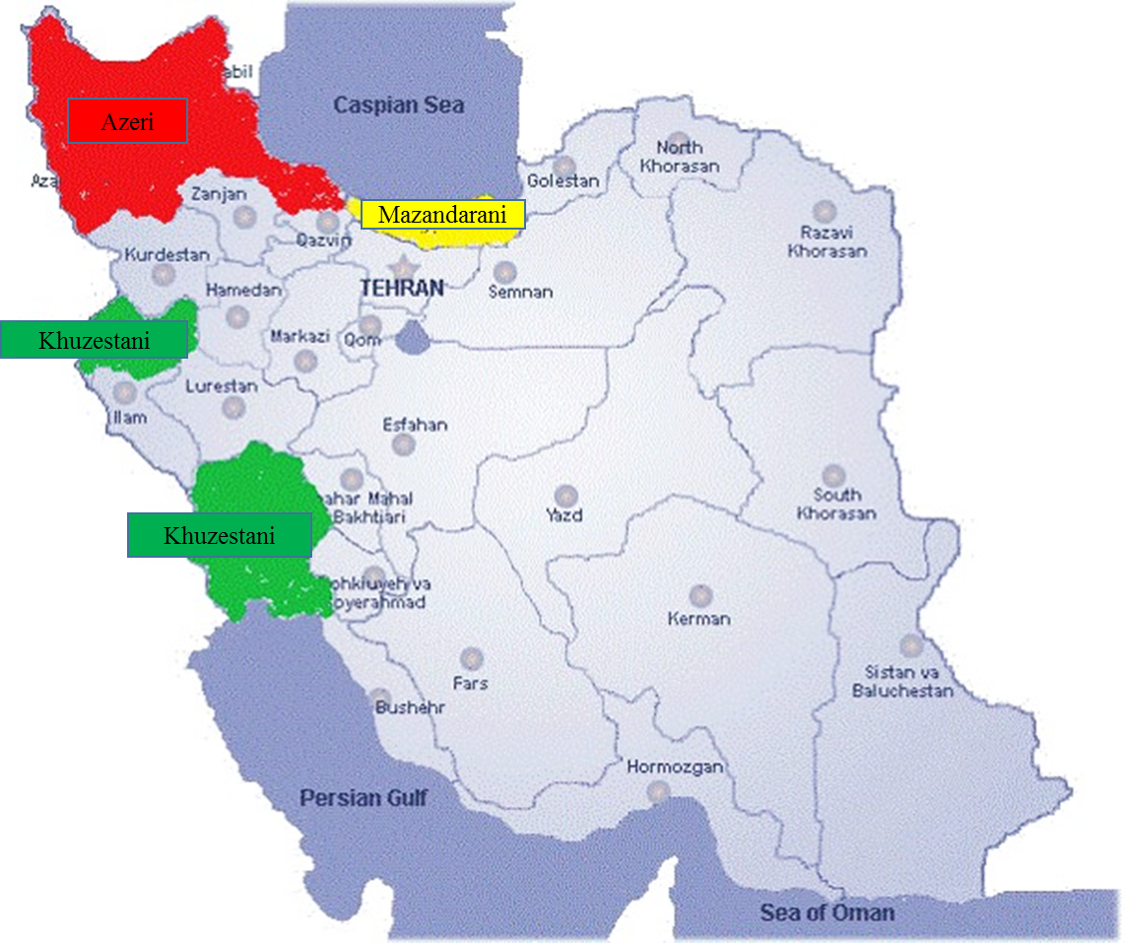

Supplement: S1 Fig — (TIF) [file pone.0217687.s001.tif]

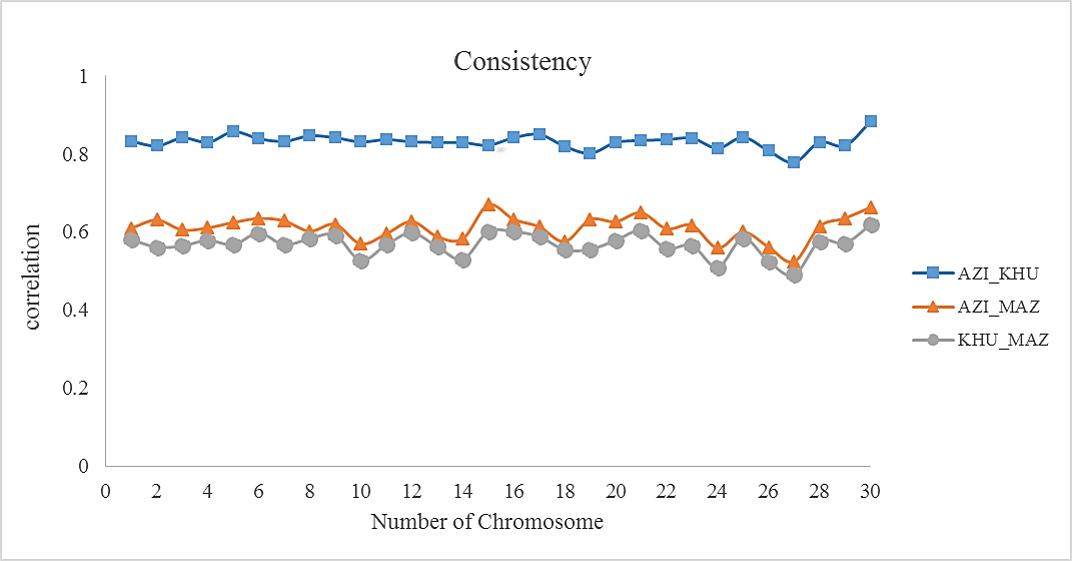

Supplement: S2 Fig — (TIF) [file pone.0217687.s002.tif]

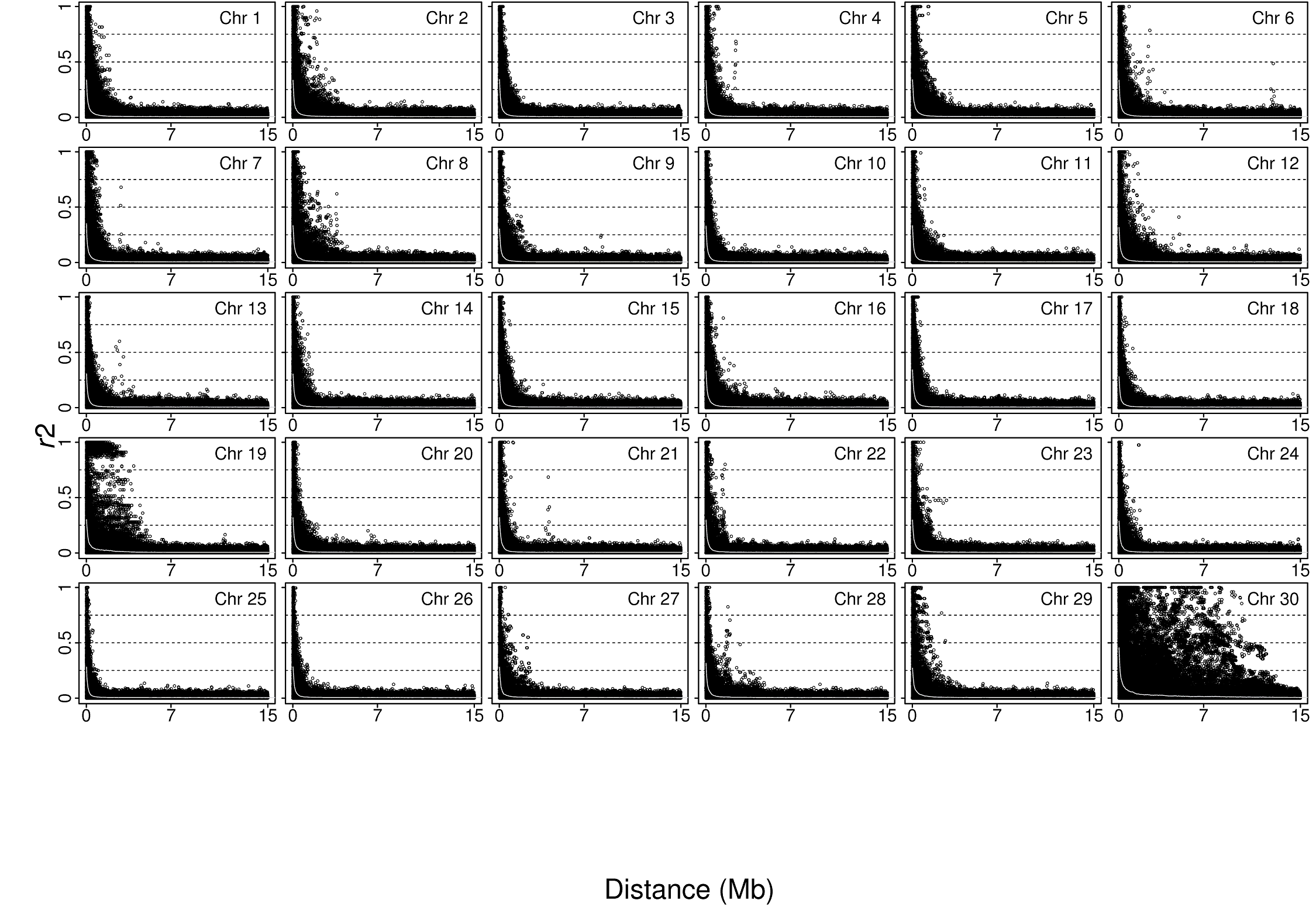

Supplement: S3 Fig — (TIF) [file pone.0217687.s003.tif]

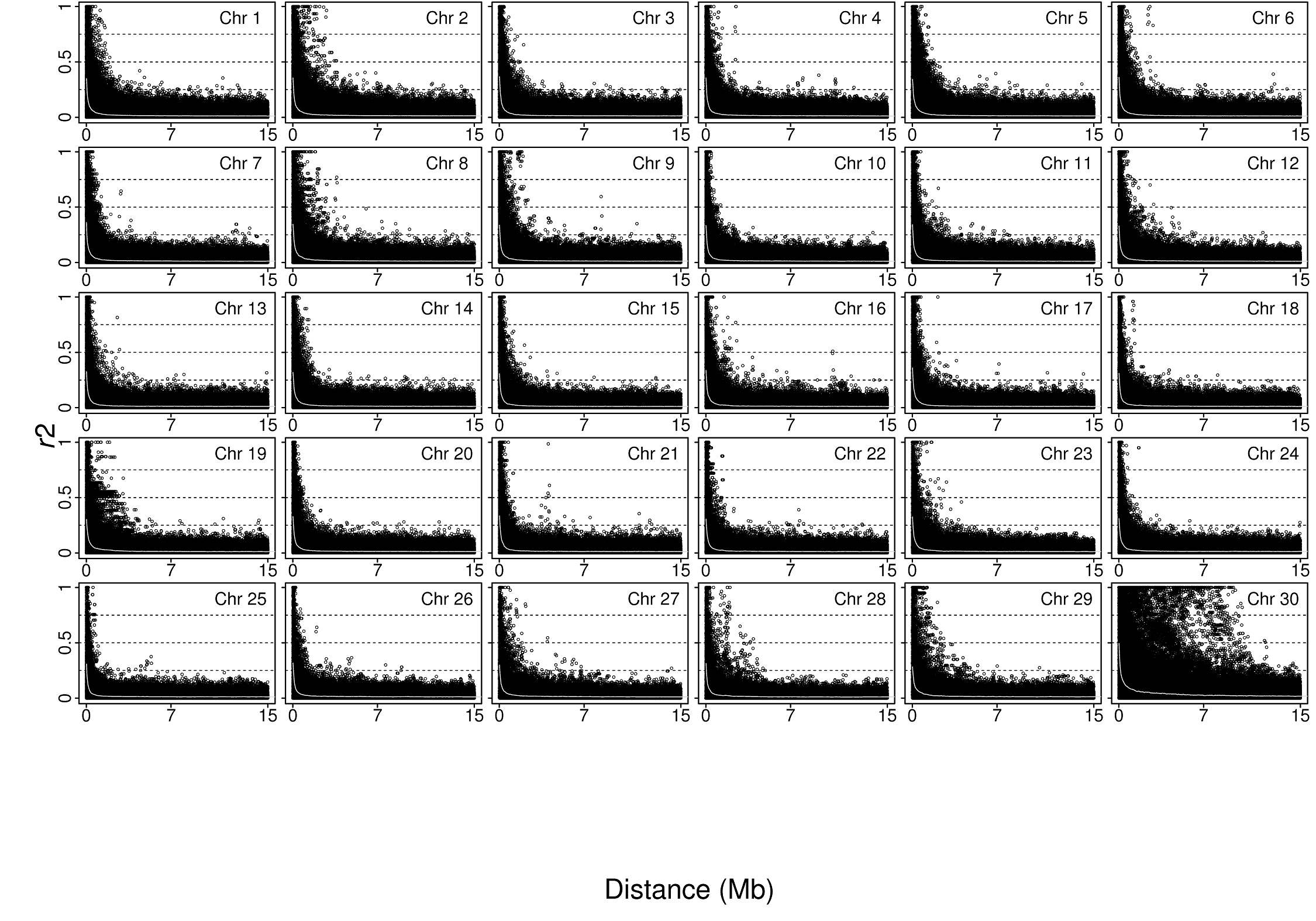

Supplement: S4 Fig — (TIF) [file pone.0217687.s004.tif]

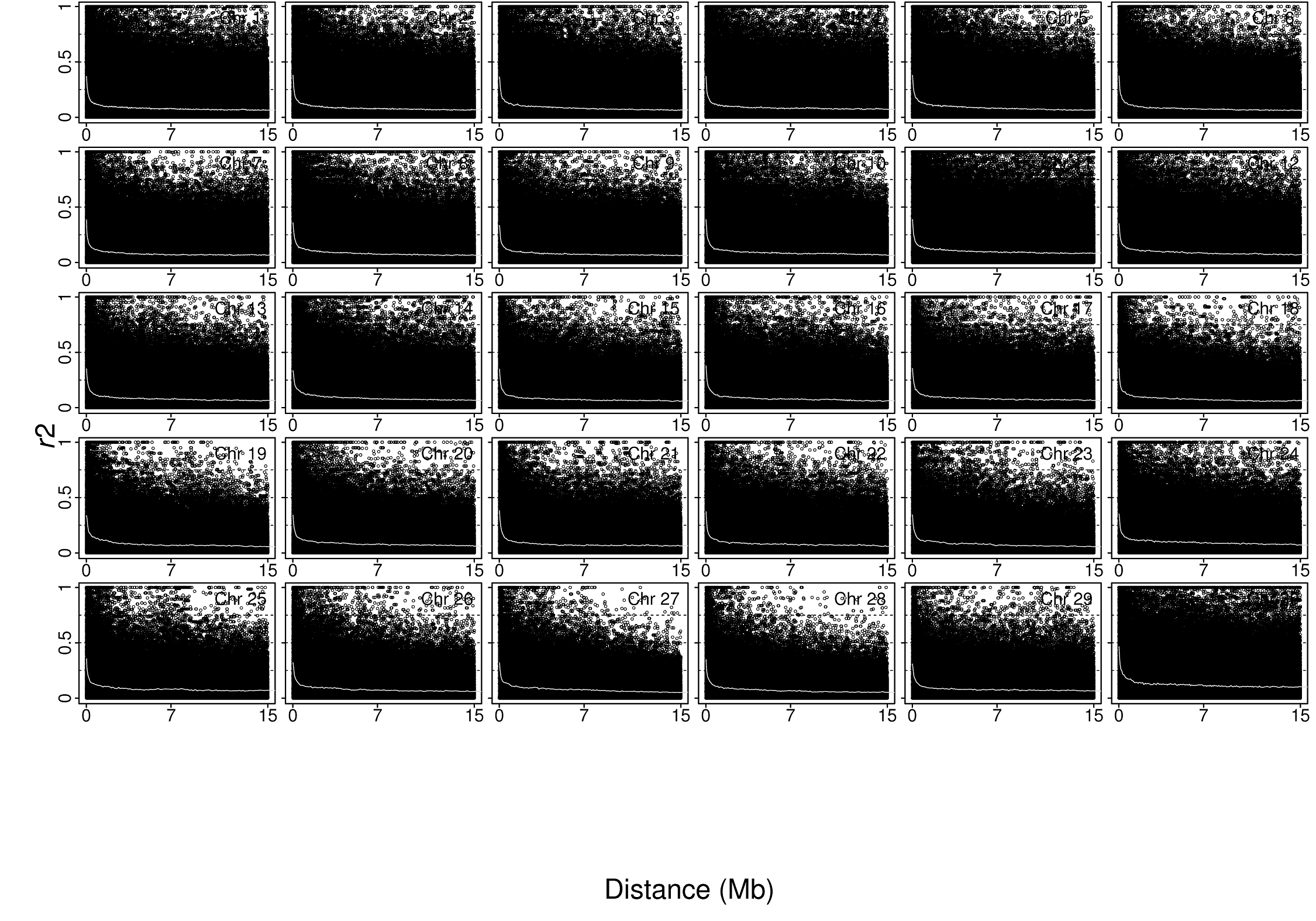

Supplement: S5 Fig — (TIF) [file pone.0217687.s005.tif]
